# Comparative Outcomes of Early Versus Delayed Ryle’s Tube Removal Following Laparoscopic Cholecystectomy: Less Tube, Less Trouble?

**DOI:** 10.7759/cureus.92615

**Published:** 2025-09-18

**Authors:** Rithu T Praveen, Krishna Prasad K, Prakash Dave, Shashirekha CA

**Affiliations:** 1 General Surgery, Sri Devaraj Urs Medical College and Hospital, Kolar, IND; 2 General Surgery, Sri Devaraj Urs Academy of Higher Education and Research, Kolar, IND

**Keywords:** cholecystectomy laparoscopic, enhanced recovery after surgery (eras), nasogastric decompression, postoperative outcomes, ryle's tube

## Abstract

Background: Nasogastric (Ryle’s) tube decompression is commonly used during laparoscopic cholecystectomy to reduce the risk of aspiration and improve visualization. However, the optimal timing of tube removal, whether intraoperative or postoperative, remains uncertain. This study evaluated early (on-table) versus delayed (≥4 hours postoperative) Ryle’s tube removal in elective laparoscopic cholecystectomy, focusing on recovery-related outcomes.

Materials and methods: A retrospective observational study was conducted at R.L. Jalappa Hospital, Kolar, involving 144 patients who underwent elective laparoscopic cholecystectomies from October 2023 to July 2025. Patients were divided into Group A (on-table removal, n = 72) and Group B (postoperative removal, n = 72). Primary and secondary outcomes included time to oral intake, postoperative nausea and vomiting (PONV), pulmonary complications, sore throat/discomfort, and hospital stay. Statistical analyses used independent t-tests and chi-square tests, with significance set at p < 0.05.

Results: Baseline characteristics were comparable between groups (p > 0.05). Group A achieved earlier oral intake (5.2 ± 1.1 hours) compared to Group B (8.4 ± 1.6 hours; p < 0.01). PONV occurred in 13 patients (18.1%) in Group A versus 27 patients (37.5%) in Group B (p = 0.02). Pulmonary complications were seen in three patients (4.2%) in Group A and seven patients (9.7%) in Group B (p = 0.18). Sore throat/discomfort was reported in 9 patients (12.5%) in Group A versus 20 patients (27.8%) in Group B (p = 0.04). The mean length of hospital stay was significantly shorter in Group A (1.8 ± 0.5 days) than in Group B (2.4 ± 0.7 days; p < 0.001).

Conclusions: Early on-table removal of the Ryle’s tube is associated with improved postoperative recovery, including earlier oral intake and reduced PONV and throat discomfort. These findings support incorporating early removal into enhanced recovery protocols for elective laparoscopic cholecystectomy.

## Introduction

Laparoscopic cholecystectomy has emerged as the gold-standard surgical treatment for symptomatic gallstone disease worldwide due to its minimally invasive nature, reduced postoperative pain, and shorter recovery time compared to open cholecystectomy [1,2]. Despite being considered a relatively straightforward and commonly performed surgical procedure, perioperative management practices continue to vary across institutions, particularly regarding the use of nasogastric decompression. Nasogastric (Ryle’s) tube insertion, a form of nasogastric intubation, is often employed intraoperatively to decompress the stomach, reduce the risk of aspiration, and improve visualization of the operative field by minimizing gastric distension [3]. Although this practice is deeply ingrained in surgical routines, there is no universal consensus on whether prolonged retention of the tube beyond the immediate intraoperative period offers measurable benefits or leads to unnecessary discomfort and complications.

Traditionally, the rationale for keeping Ryle’s tube in situ for several hours postoperatively is rooted in concerns over postoperative nausea and vomiting (PONV), aspiration risk, and delayed gastric emptying following anesthesia [4]. Postoperative ileus and GI dysfunction are well-recognized contributors to delayed recovery in abdominal surgery. Hence, many surgeons continue to retain the tube for a few hours post-surgery, assuming it prevents gastric stasis and minimizes the likelihood of aspiration. However, growing evidence suggests that these benefits may be overestimated, particularly in minimally invasive procedures such as laparoscopic cholecystectomy, which are associated with less postoperative ileus compared to open surgeries [5]. Furthermore, unnecessary prolongation of nasogastric intubation is linked with adverse outcomes, including patient discomfort, sore throat, impaired swallowing, epistaxis, prolonged initiation of oral feeding, increased risk of aspiration from tube misplacement, and even respiratory complications due to impaired airway clearance [6,7].

In recent years, the Enhanced Recovery After Surgery (ERAS) protocols have revolutionized perioperative care by advocating for interventions that expedite recovery, reduce complications, and enhance patient satisfaction [8]. A key recommendation of ERAS guidelines is the avoidance of unnecessary tubes and drains, including the early removal of Ryle’s tubes. Early removal, or better yet, avoidance of postoperative Ryle’s tube retention, has been associated with earlier initiation of oral intake, shorter hospital stays, and improved patient comfort in various GI surgical procedures [9]. However, adherence to ERAS principles remains inconsistent, particularly in resource-constrained settings and in surgeries where traditional practices dominate decision-making. In India and several other low- and middle-income countries, the delayed removal of Ryle’s tubes following laparoscopic cholecystectomy continues to be widely practiced, largely due to cultural inertia in surgical training and perceived safety concerns [10].

The novelty of our study lies in its context and applicability. While numerous investigations have evaluated Ryle’s tube use in GI surgeries broadly, few have focused specifically on laparoscopic cholecystectomy, which constitutes one of the most commonly performed procedures in general surgery. Moreover, there is limited literature from India examining how modifications in perioperative practices, such as Ryle’s tube management, can influence recovery outcomes. Given that laparoscopic cholecystectomy accounts for a significant surgical burden in India, insights from this study are not only academically relevant but also hold practical significance in shaping national perioperative care protocols. Therefore, this study aims to evaluate the impact of early (on-table) versus delayed (≥4 hours postoperative) Ryle’s tube removal in patients undergoing elective laparoscopic cholecystectomy at a tertiary care center in India.

## Materials and methods

The present study was designed as a retrospective observational study and was conducted at R.L. Jalappa Hospital and Research Centre, Kolar, Karnataka, India. Patient case records maintained in the Department of Surgery were systematically reviewed for a period of nearly two years, from October 2023 to July 2025. The study design was chosen because retrospective data allowed for the inclusion of a larger number of patients across a defined timeframe, while also ensuring feasibility and minimization of resource constraints. Patient records were carefully examined to identify those who satisfied the inclusion criteria and to exclude those who did not meet eligibility requirements, to generate a homogenous study population. A predefined, well-structured framework of inclusion and exclusion criteria was followed to reduce bias and potential confounding factors that could affect the outcomes. The study was approved by the Central Ethics Committee of Sri Devaraj Urs Academy of Higher Education and Research (approval number: SDUAHER/R&D/CEC/SDUMC/PG/170/NF-2025-26).

Inclusion criteria

Patients were eligible for inclusion if they were aged between 18 and 70 years and had undergone a planned elective laparoscopic cholecystectomy during the study period. To minimize perioperative variability, only patients belonging to the American Society of Anesthesiologists (ASA) physical status grades I or II were included. This ensured the population consisted of relatively healthy individuals without significant systemic illness, thereby reducing the influence of comorbidities on the primary and secondary outcomes. An additional key inclusion requirement was the availability of detailed and well-documented operative and postoperative records, particularly concerning the timing of Ryle’s tube removal. This was essential to ensure that each patient could be reliably categorized into the appropriate study group and that outcomes were measured accurately.

Exclusion criteria

The exclusion criteria were intentionally comprehensive to minimize heterogeneity and to avoid introducing clinical conditions that might confound the interpretation of results. Patients undergoing emergency laparoscopic cholecystectomy or cases that required conversion to open cholecystectomy at any point were excluded. Incomplete operative or postoperative records, especially concerning perioperative events and Ryle’s tube removal details, also led to exclusion. Patients with pre-existing upper GI disorders, such as gastric outlet obstruction or gastroesophageal reflux disease, were omitted, as these conditions could independently predispose to nausea, vomiting, or aspiration, thereby confounding outcomes. Similarly, patients with severe systemic comorbid illnesses or those who were immunosuppressed were excluded since these factors might adversely influence recovery parameters, increase postoperative complications, and interfere with meaningful group comparisons.

Group classification

The central differentiating factor for classification into groups was the timing of Ryle’s tube removal following laparoscopic cholecystectomy. Group A (Early Removal Group): This group included patients whose Ryle’s tube was removed intraoperatively, shortly after completion of the surgery, while the patient was still in the operating room. Group B (delayed removal group): This group consisted of patients in whom the Ryle’s tube was removed at least four hours after surgery, usually following stabilization in the immediate postoperative recovery period. This classification allowed the study to evaluate whether early removal, as suggested by ERAS protocols, provided any measurable benefit compared to the more traditional approach of delayed removal.

Sampling method and sample size

**Figure 1:**
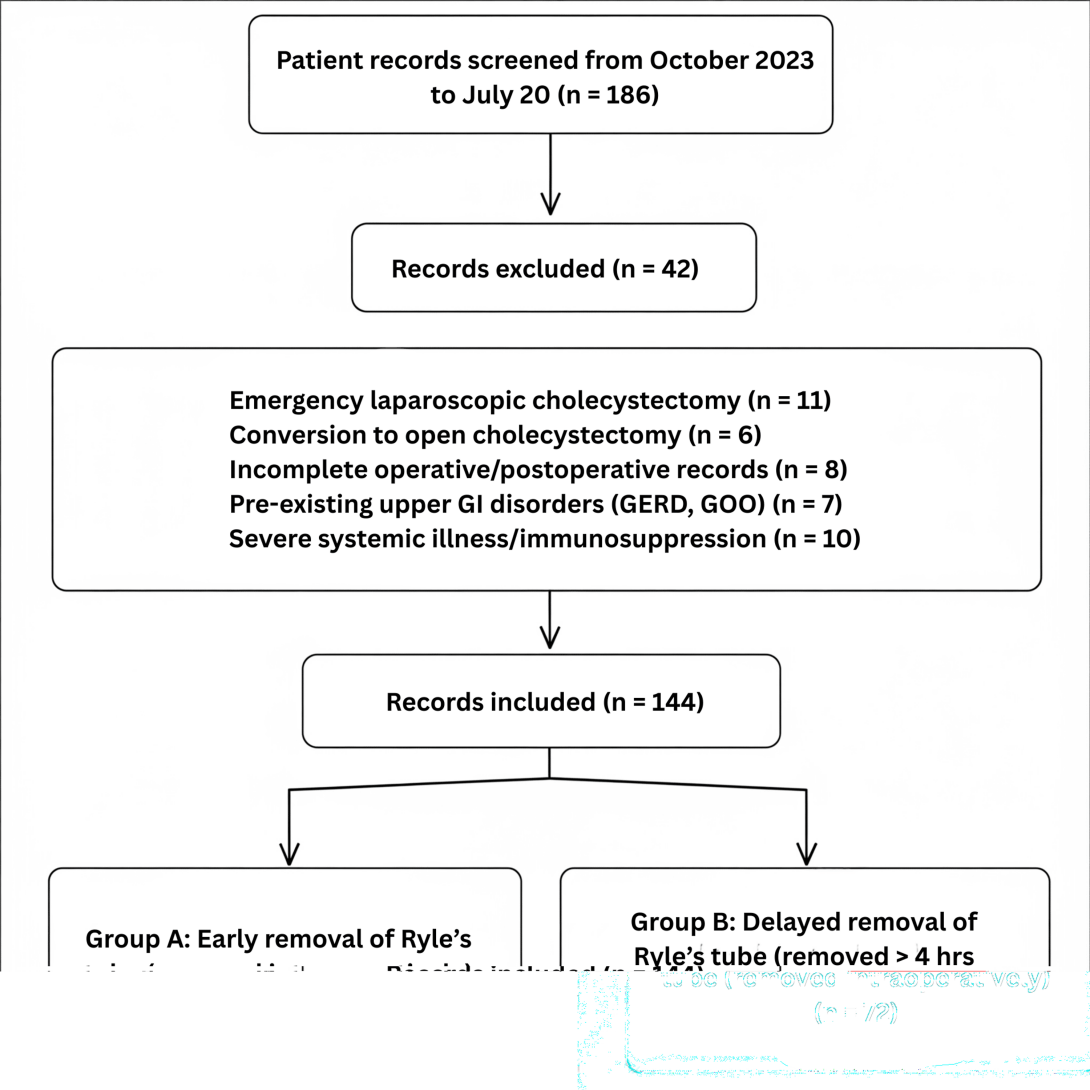
Flow diagram showing participant selection GI: gastrointestinal, GERD: gastroesophageal reflux disease, GOO: gastric outlet obstruction

For this study, a convenience sampling strategy was used. All patients who met the provided inclusion and exclusion criteria during the two years of the study were included. All patients undergoing elective laparoscopic cholecystectomy during the study period were initially screened using the operating theater and admission registers. Their medical records were reviewed to assess eligibility based on predefined inclusion (elective cases, age >18 years, ASA I-II) and exclusion criteria (emergency surgeries, intraoperative complications, conversion to open surgery, or pre-existing GI conditions). After preliminary screening and evaluation of the available documentation for 186 participants, 144 patients were recruited, with 72 individuals allocated to each group to facilitate comparative analysis. The flow diagram of the study is depicted in Figure 1.

Data collection parameters

Primary and secondary outcome measures were taken from patient medical records. Some of the key variables were the duration of the first oral intake following surgery and PONV. Other outcomes measured were the occurrence of pulmonary complications, including atelectasis or aspiration pneumonia, postoperative sore throat or tube discomfort, and the total hospital stay from admission until discharge.

Statistical analysis

Data were analyzed using SPSS Statistics version 25 (IBM Corp., Released 2017. IBM SPSS Statistics for Windows, Version 25.0. Armonk, NY: IBM Corp.). Continuous variables were summarized by the calculation of means and standard deviations, whereas categorical variables were summarized in frequencies and percentages. To compare continuous variables between the two groups, either the independent t-test or the Mann-Whitney U test was utilized based on the nature of the distribution of the data. Categorical variables were compared using the chi-square test or Fisher's exact test, as appropriate. A p-value of less than 0.05 was regarded as statistically significant for all the tests that were carried out.

## Results

In Table 1, a total of 144 patients were included in the study, with Group A (on-table Ryle’s tube removal) and Group B (postoperative removal) each comprising 72 participants. The mean age was 42.6 ± 10.2 years in Group A and 44.1 ± 11.3 years in Group B (p = 0.48). Among Group A, 40 (56.0%) were male and 32 (44.0%) were female, while Group B had 37 (51.4%) males and 35 (48.6%) females (p = 0.68). Regarding ASA grade, 52 (72.2%) participants in Group A and 50 (69.4%) in Group B were classified as Grade I, while 20 (27.8%) in Group A and 22 (30.6%) in Group B were classified as Grade II (p = 0.81).

**Table 1:** Baseline characteristics of the study participants (n = 144) * chi-square test, # independent sample t-test, p < 0.05 is statistically significant ASA: American Society of Anesthesiologists, SD: standard deviation

Variable	Group A = 72 n (%)	Group B = 72 n (%)	Test statistic	p-value
Age (years, mean ± SD)	42.6 ± 10.2	44.1 ± 11.3	t = 0.71	0.48#
Gender				
Male	40 (56.0%)	37 (51.4%)	χ² = 0.26	0.68*
Female	32 (44.0%)	35 (48.6%)		
ASA grade				
I	52 (72.2%)	50 (69.4%)	χ² = 0.73	0.81*
II	20 (27.8%)	22 (30.6%)		

 In Table 2, the mean time to oral intake was significantly shorter in Group A (5.2 ± 1.1 hours) compared to Group B (8.4 ± 1.6 hours, p < 0.001). PONV occurred in 13 patients (18.1%) in Group A versus 27 patients (37.5%) in Group B (p = 0.02). Pulmonary complications were observed in three patients (4.2%) in Group A and seven patients (9.7%) in Group B, though this difference was not statistically significant (p = 0.18). Sore throat or discomfort was reported in nine patients (12.5%) in Group A compared to 20 patients (27.8%) in Group B (p = 0.04). The mean length of hospital stay was significantly shorter in Group A (1.8 ± 0.5 days) than in Group B (2.4 ± 0.7 days, p < 0.001).

**Table 2:** Comparison of postoperative outcomes among both the study groups (n = 144) * chi-square/Fisher's exact test, # independent sample t-test, p < 0.05 is considered significant SD: standard deviation

Outcome	Group A n (%)	Group B n (%)	Test statistic	p-value
Time to oral intake (hours, mean ± SD)#	5.2 ± 1.1	8.4 ± 1.6	t = 13.1	<0.001#
Postoperative nausea/vomiting	13 (18.1%)	27 (37.5%)	χ² = 5.6	0.02*
Pulmonary complications	3 (4.2%)	7 (9.7%)	χ² = 1.7	0.18*
Sore throat/discomfort	9 (12.5%)	20 (27.8%)	χ² = 4.2	0.04*
Length of hospital stay (days, mean ± SD)#	1.8 ± 0.5	2.4 ± 0.7	t = 5.3	<0.001#

## Discussion

This study demonstrates that early removal of Ryle's tube after laparoscopic cholecystectomy is associated with improved postoperative recovery, as evidenced by an earlier return to oral diet, lower incidences of nausea and vomiting, and shorter hospital stays. These results are consistent with the principles of ERAS, which support minimal use of invasive equipment to facilitate faster functional recovery.

Increasing evidence suggests that routine postoperative nasogastric decompression is not only unnecessary but may even be a barrier to recovery. A seminal meta-analysis by Cheatham et al. (1995) revealed that selective Ryle's tube use (versus routine use), as opposed to routine placement, had fewer respiratory complications and earlier return of bowel function following elective laparotomy [9]. Nelson et al. (2005) also conducted a systematic review, which revealed no significant benefit in postoperative outcomes with routine nasogastric decompression. Still, patients without routine Ryle’s tube insertion experienced earlier bowel movement and less discomfort [10].

Specific to bowel anastomosis, Negi et al. (2019) conducted a randomized controlled trial of early Ryle’s tube removal versus the standard prolonged retention. They found that early removal was associated with earlier return of bowel activity, earlier return of oral intake, and reduced postoperative ileus, without an increase in complications [11]. These advantages, which we see in our study, again prove the principle that early Ryle's tube removal is a safe and effective procedure. Other studies agree with this result in more complex abdominal operations. Shamil et al. (2010) and Chuslip et al. (2021) found that nasogastric decompression did not reduce postoperative complications, suggesting that the omission or earlier removal of Ryle’s tubes should be the standard in elective surgery [12,13].

The pattern of reduced pulmonary complication rate in the early removal group in our study, albeit not statistically significant, is consistent with the literature. Sapkota and Bhandari (2013) illustrated that inappropriate Ryle’s tube insertion after emergency laparotomy had no protective benefit and could be associated with higher respiratory morbidity [14]. Weijs et al. (2017), in a systematic review of patients who underwent esophagectomy, found no added safety with prolonged Ryle’s tube use, presenting evidence for the generalizability of early removal to additional procedures [15]. Bauer also obtained the same result, with patients who did not have postoperative Ryle’s tubes experiencing less throat pain and a faster recovery [16]. The increased incidence of sore throat in the delayed group is because of mechanical irritation secondary to prolonged nasogastric intubation. This concurs with the study of Ullah et al. (2017), which proved greater discomfort and throat complaints in patients with prolonged Ryle’s tube use after upper GI surgery [17].

In addition, pediatric literature also affirms the safety of early removal. Dinsmore et al. (1997) and Chusilp et al. (2021) both agreed that Ryle’s tube decompression is not required even after primary intestinal operations in children, reiterating that physiological recovery does not rely on the length of tube placement [13,18]. Our findings are also supported by MacRae et al. (1992), who reported that routine Ryle's tube removal after GI surgery did not lead to an increase in morbidity and allowed for earlier recovery [19].

While these are positive findings, the current study is limited as well. As a retrospective observational study, it naturally involves some methodological limitations. For one, data were not gathered in real-time but retrieved from available medical records, which can potentially introduce errors or inconsistencies based on incomplete documentation or subjective recording by various clinicians. Second, retrospective designs cannot control variables, thereby heightening the possibility of confounding variables that would affect outcomes, such as variations in intraoperative technique, anesthetic regimens, or standards of postoperative care that were not uniform or controlled for.

Despite ERAS guidelines, Ryle's tubes were kept postoperatively in a portion of this Indian cohort due to persistent traditional surgical practices and a cautious approach. Surgeons often delayed removal due to ingrained fears of postoperative nausea, vomiting, and aspiration, prioritizing perceived safety over comfort. The study notes that formal ERAS protocols for laparoscopic cholecystectomy are not universally adopted in India, leading to variability based on individual surgeon preference. This highlights the gap between international evidence and local real-world practice, which this study aims to bridge by providing institution-specific data to encourage protocol change.

Since this is a single-center trial, generalizability may also be limited because patterns of practice and postoperative care guidelines might differ institutionally. While objective end points like nausea, vomiting, and hospital stay were documented, patient-rated end points like discomfort, bloating, or satisfaction were not measured. The inclusion of such subjective endpoints might better define the advantage of early Ryle's tube removal in subsequent prospective studies. However, this study contributes to the increasing evidence that routine or extended nasogastric decompression is not required following laparoscopic cholecystectomy. Early removal of the tube not only saves the patient from distress but also allows quicker postoperative recovery without increasing the risk of complications.

## Conclusions

Early (on-table) removal of Ryle’s tube after laparoscopic cholecystectomy was associated with several favorable postoperative outcomes, including a significantly earlier return to oral intake, reduced incidence of postoperative nausea and sore throat, and a shorter duration of hospital stay, without any increase in pulmonary complications. These findings support the safety and efficacy of early tube removal, challenging the traditional practice of prolonged postoperative retention. Incorporating early removal into ERAS protocols for elective cholecystectomy could enhance patient comfort, reduce morbidity, and optimize resource utilization through faster recovery and reduced hospital stay. Adoption of such evidence-based modifications in perioperative care pathways may improve overall surgical outcomes and patient satisfaction, while also contributing to cost-effective healthcare delivery.
